# Evaluating the Effectiveness of Education Support Programs for Hospitalized Students With Chronic Health Conditions: Protocol for a Feasibility Study of a Controlled Trial

**DOI:** 10.5334/cie.10

**Published:** 2020-11-20

**Authors:** Tony Barnett, Sharon Goldfeld, Margaret Kelaher

**Affiliations:** 1 University of Melbourne, AU; 2 Murdoch Children’s Research Institute, AU

**Keywords:** Chronic illness, education, child development, schools, pediatric hospitals

## Abstract

Chronic health conditions in children and young people can have a significant impact on their ability to maintain engagement in school, education, and learning. While this functional limitation affects only about 1.6% of all children and young people, the absolute number is not inconsequential. In Australia, for example, the number is in the order of 67,000 children and young people. Furthermore, research has shown that this group of young learners are at increased risk of lower academic, social, and emotional and quality of life outcomes than their healthy peers, both in the short and the longer term. For this reason, most pediatric hospitals in western developed countries have hospital-based schools that aim to prevent children and young people with a chronic health condition from disengaging from school, education, and learning. However, there is a lack of robust evidence of the effectiveness of these education support programs. This protocol for a feasibility study of the effectiveness of evaluating an education support program in Australia aims to identify a priori the methodological key features of a robust trial, including developing an answerable research question, choosing a controlled study design that compares the outcomes of both an intervention group and a well-matched non-intervention or control group, eligibility criteria, important and validated outcome measures such as quality of life, and how statistical data should be analyzed and reported. Lessons learned from the proposed feasibility study will be used to inform a larger-scale study.

Pediatric chronic health conditions are defined as illnesses that have lasted, or are expected to last, at least six months; have a pattern of recurrence or deterioration; have a poor prognosis; and produce consequences or sequelae that impact on the individual’s quality of life (Australian Institute of Health and Welfare [AIHW], 2005; Martinez & Ercikan, 2009). Common childhood chronic health conditions include cancer, cystic fibrosis, Crohn’s disease, diabetes, epilepsy, and asthma. Approximately 20%–30% of children and young people are reported to have a chronic health condition, depending on the definition used. A relatively small percentage (1.6%) have a chronic health condition that is severe enough to impact on their functional ability to regularly attend school (Shaw & McCabe, 2008). In Australia this amounts to around 67,000 students.

For this reason, most pediatric hospitals in western developed countries have hospital-based schools or education support services. For example, the Children’s Hospital School at Great Ormond Street Hospital, London, aims to minimize the interruption and disruption to children and young people’s education so that academic progress and an interest in learning will continue, as far as medical circumstances permit. Similarly, at the Hasbro Children’s Hospital, Rhode Island, in the United States, the hospital school has the following aims: to make the transition from hospital back to school as smooth as possible for the patient and classmates, to maintain academic involvement with the patient’s home school and teachers, and to utilize hospitalization as a unique and positive learning experience. And in Melbourne, Australia, teachers at the Education Institute at the Royal Children’s Hospital work with more than 2,000 school-aged students each year to keep them engaged with their education and connected to their regular school and classmates.

The rationale for these education support programs is that children and young people with chronic health conditions are at increased risk of disengagement from school, education, and learning, and, worse, academic, social, and emotional and quality of life outcomes both in the short and the longer term (Martinez & Ercikan, 2009; Maslow, Haydon, McRee, & Halpern, 2012; Nasuuna, Santoro, Kremer, & Silva, 2016). Furthermore, access to a quality education for all children, including those managing a chronic health condition, is a right that is enshrined in both national and international laws (UNICEF, 2006).

While the common goal of education support programs is to prevent students with a chronic health condition from disengaging from school, education, and learning and to maintain continuity in their human development processes (Seymour, 2004), many different types of services or interventions exist for this group of students, shaped by their setting and context, and described differently (Dempsey, 2019). Nonetheless, there appear to be some common elements or themes to these programs (Capurso & Dennis, 2017). The description of the education support program at the Queensland Children’s Hospital, Australia, funded by the State Department of Education, reflects many of the key common themes of education support programs, as described by Capurso and Dennis (2017). They are:

1. Students have access to learning at all stages of their illness.
2. Staff deliver high-quality teaching and learning programs.
3. Students experience continuity of learning.
4. Students and staff feel a sense of belonging to their school communities.
5. Staff engage in multidisciplinary collaboration.

According to Bond (2019), each of these main themes may be further broken down into a series of variables that render the five key elements effective, as shown in Figure 1.

**Figure 1:**
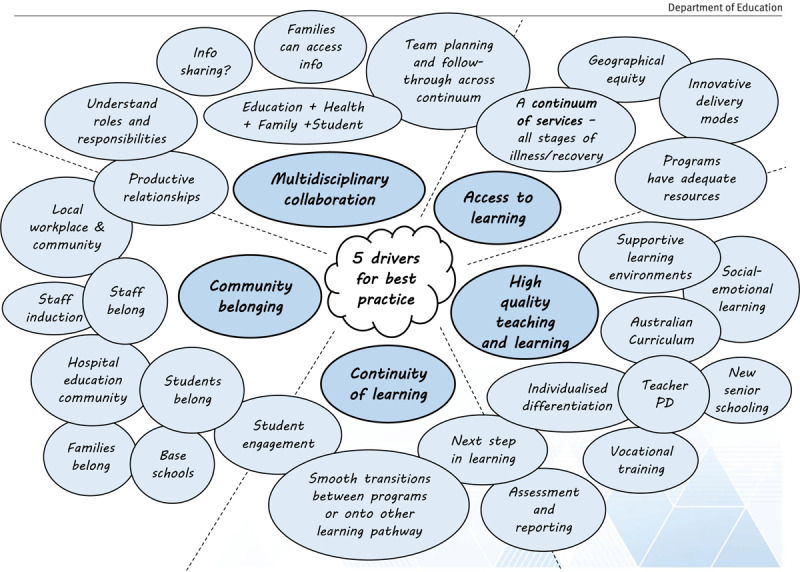
Best practice in education support for hospitalized students in Queensland, Australia.

However, there is a need to generate more robust evidence about the effectiveness of these types of education support programs. A systematic review of educational support services for children and adolescents with chronic health conditions (Barnett, 2018) found just four controlled studies of a diverse range of education support programs aimed mostly at children with cancer, and inconclusive evidence about their effectiveness. It also revealed that the current state of the evidence about the effectiveness of these interventions is predominantly made up of qualitative research, case studies, and expert opinion, which are known to be more affected by bias than more rigorous controlled studies that include both an intervention group and a comparison or control group (Chalmers, 2005).

## The Present Study

To fill this gap, the purpose of this paper, therefore, was to consider how a more rigorous controlled study of the effectiveness of education support services for students with chronic health conditions could be undertaken. Specifically, it outlines the protocol for a feasibility study as a first stage to evaluating the effectiveness of these programs. Feasibility studies are considered useful as preparation for the conduct of a larger controlled evaluation of complex interventions (Thabane et al., 2016), with the goal of reducing uncertainty and thereby increasing the chance of a successful larger study (Thabane et al., 2016). The proposed protocol is based upon and includes recommended and relevant sections of the Standard Protocol Items: Recommendations for Interventional Trials (SPIRIT) checklist (Chan, Altman, Dickersin, & Moher, 2013).

Specifically, the aims of the feasibility study are to:

1. Examine the effectiveness of an education support program for school-aged students with chronic health conditions on their engagement in education and learning, and quality of life;
2. Trial the recruitment and retention of study participants;
3. Identify and trial the use of validated outcome measures;
4. Obtain feedback from stakeholders about the feasibility of the above with the view of conducting a larger scale trial.

## Method

### Study Design and Setting

Many researchers (e.g., Chalmers, 2005; Gray, 2001; Littell & Shlonsky, 2010; Shlonsky & Gibbs, 2004) consider the randomized controlled trial (RCT) the “gold standard” in effectiveness evaluations as it is viewed to be the best design for minimizing the influence of bias. But for many others in the pedagogical community, the debate on this point is still open (Biesta, 2007). In our circumstance, given that access to a quality education is a right of the child along with the potential public sensitivity of randomly allocating hospitalized children and young people to either receive education support or not while in hospital, an RCT may not be considered either appropriate or ethical.

Fortunately, other controlled study designs are available for consideration. Controlled studies (i.e., studies with both an intervention group and a well-matched non-intervention or control group) are considered best for evaluating the effectiveness of an intervention because they state the counterfactual. That is, they investigate and compare both what happens if a group of people receive the intervention and what happens if a group of people don’t receive the intervention. This, in turn, allows stronger and more robust claims of causality and effectiveness of a given intervention; that is, that any differences in results between the two groups were due and can be attributed to the intervention (Chalmers, 2005; Shlonsky & Gibbs, 2004).

One such design is that of a controlled cohort study, where both the intervention group and the control group exist “naturally” in the community. Such is the case in Victoria, Australia, where the Department of Education and Training (DET) funds education support programs at some, but not all, pediatric hospitals and departments. The DET funds education programs at two pediatric hospitals (Royal Children’s Hospital and Monash Children’s Hospital), but not four regional pediatric departments in hospitals in Warrnambool, Ballarat, Gippsland, and La Trobe. Children and young people with chronic illnesses admitted to the latter hospitals where there is no funded education support program, therefore, naturally form the control or comparison group.

The subjects for the current study consisted of the children and young people under treatment for a chronic health condition at the Royal Children’s Hospital (the intervention group) and the four regional hospitals.

### Developing an Answerable Research Question

An important first step in effectiveness evaluations is to develop an answerable research question in PICO format (Shlonsky & Gibbs, 2004). The general and overarching aims of education support programs have been discussed earlier. As such, the specific research question for our evaluation of the effectiveness of education support programs in Victoria, Australia, is as follows.

**Table:** 

Population (P)	Do hospitalized children with a chronic health condition,
Intervention (I)	who receive hospital-based education support
Comparison (C)	as compared to those who receive no hospital-based education support,
Outcomes (O)	have higher levels of engagement in education and learning and quality of life?

### Participants

Participants will need to meet the following eligibility criteria:

- Being school aged (5–17 years old)
- Having a chronic illness (for example, but not limited to, cancer, cystic fibrosis, eating disorder and Crohn’s disease/renal failure)
- Having an expected length of stay (LOS) in hospital of five or more days in a six-month period

The criterion of an expected LOS of 5+ days was informed by the work of Prof. Stephen Zubrick and colleagues, whose Australian research investigated the impact of school absence on academic performance. Their study (Hancock, Shepherd, Lawrence, & Zubrick, 2013) was based on students who were enrolled in the public school system in Western Australia from 2008 to 2012. The data included information collected by schools on students and their caregivers upon enrolment, attendance records, and the results of the National Assessment Program – Literacy and Numeracy (NAPLAN). The authors hypothesised a threshold effect of absence from school on academic performance, such that a small amount of absence from school might have minimal effect on academic performance, but beyond some threshold attendance level there would be a noticeable drop in measured academic performance. Their findings, however, generally support the notion that “every day counts.” That is, every day of absence from school is associated with progressively lower achievement in numeracy, writing, and reading.

They also found that, generally, an absence of four weeks per half year resulted in performance dropping to that of the national minimum standard. However, school absence had a much greater influence on the achievement of students in lower socio-economic index schools with absences of three weeks per half year resulting in performance dropping to that of the national minimum standard.

In earlier research on categories of at-risk attendance, again in the Western Australian school jurisdiction and based on empirical findings from the 1993 Western Australian Child Health Survey (WACHS), Zubrick, Silburn, Gurrin, and Shepherd (1997) found that students who were, on average, absent for two weeks per half year would be considered to be at-risk for lower academic competence.

Furthermore, in 2014 the Royal Children’s Hospital Education Institute undertook an investigation into what happens when the young people/students they had worked with were discharged from hospital. Results showed that one month after discharge, one in three children were still at home and had not returned to school. Of the children who had returned to school during the month after discharge, two out of three spent a period of time at home prior to returning to school – the average length of time spent at home prior to returning to school was 13 days (ARACY, 2015).

For these reasons, in this feasibility study, the eligibility criterion of an expected in-hospital LOS of 5 or more days in a six-month period was chosen as a proxy measure of an expected total length of absence from school of potentially 14 (or more) days in a six-month period. Such a length of absence would put the child in an “at-risk” category of worse academic outcomes.

### Intervention to Be Tested

The education support program at the Royal Children’s Hospital (RCH) in Australia is funded by the Victorian DET and employ qualified teachers to work with hospitalized young people/students to help maintain engagement with the students’ regular schools and peers, and engagement in education and learning. The Education Institute teachers evaluate new patients to determine the appropriate level of educational support. Evaluations are based on need or educational risk, with eligible students being offered individual or group learning sessions, or a combination of both. Each student receives an Individual Learning Plan (ILP) prepared in collaboration with the student, families/carers, and the enrolled educational setting. The ILP is reviewed regularly and updated in line with the student’s educational progress. The hospital kindergarten program is led by qualified early childhood educators who, through engaging play-based learning, help prepare children for school. To be eligible for the program, children must be at least 4 years old before April 30 in the year of enrolment. The Education Institute offers an evidence-based program built on high-impact teaching strategies, with a focus on literacy and numeracy underpinned by the Victorian Curriculum.

**Figure 2:**
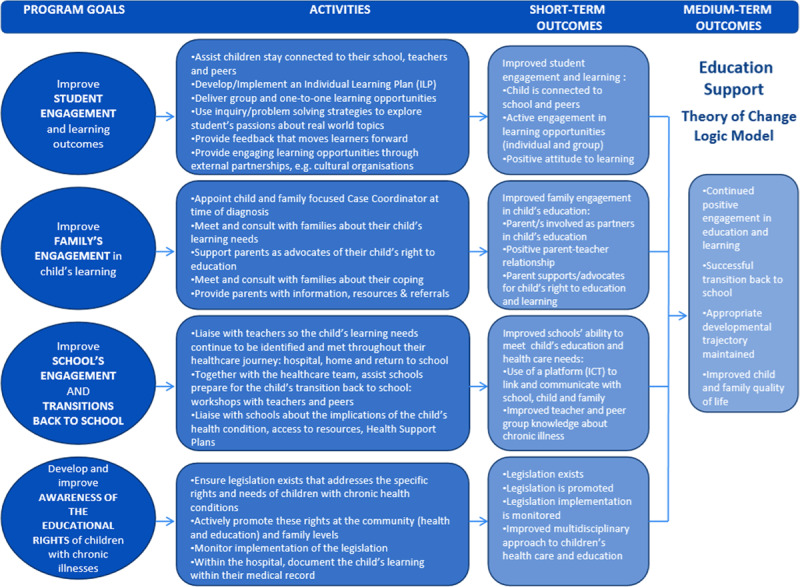
Logic Model of an Education Support Program for Children and Young People with a Chronic Health Condition.

Elements of the education support program are described in a program logic model developed in consultation with staff from the Centre for Program Evaluation at the University of Melbourne. It depicts a socio-ecological approach that recognizes the influence of a number of spheres within which the child functions on a daily basis (Bronfenbrenner, 1995). These include the spheres or levels of the individual, family, schools, community and service systems. (For more detail, see Figure 2: Logic Model of an Education Support Program for Children and Young People with a Chronic Health Condition; Barnett, 2018.) The key activities and variables of the intervention across the first three spheres are listed in Table 1. Finally, the education support model at the RCH also reflects the five common themes of pediatric hospital-based education support programs as described in the introduction.

**Table 1:** Key activities of education support programs for hospitalized students with chronic health conditions.

Sphere of influence/level	Key activities of education support

Individual young person	Assist children stay connected to their school, teachers and peers Develop/Implement an Individual Learning Plan (ILP) Deliver group and one-to-one learning opportunities Use inquiry/problem solving strategies to explore student’s passions about real world topics
Family	Meet and consult with families about their child’s learning needs Support parents as advocates of their child’s right to education Meet and consult with families about their coping Provide parents with information, resources & referrals
School	Liaise with teachers so the child’s learning needs continue to be identified and met throughout their healthcare journey: hospital, home and return to school Together with the healthcare team, assist schools prepare for the child’s transition back to school: workshops with teachers and peers

### Ethics

Ethics approval will be obtained for the study from a registered Australian Human Research Ethics Committee prior to commencement of the research.

### Recruitment and Consent

Approval to undertake the feasibility study will be sought from the Victorian DET. Once such approval has been obtained, all five pediatric sites (one pediatric hospital as the intervention site and four regional hospitals with pediatric wards as the control sites) will be approached by the lead researcher and invited to participate in the feasibility study. Once consent has been obtained from these two levels, we will proceed with the rolling recruitment of individual young people. Specifically, all new inpatients as they present to hospital and who meet the eligibility criteria will be approached by the lead researcher and asked if they would participate in the trial. Both the young person and his or her parent/s or caregiver/s will receive a Plain Language Statement about the study and asked to provide written consent.

For aim number 4 (Obtain feedback from stakeholders about the feasibility of the above with the view of conducting a larger scale trial), we will identify two key stakeholders at each of the five locations and ask them to participate in semi-structured interviews with the lead researcher. The purpose of the interviews will be explained, and verbal consent will be obtained and recorded by the lead researcher.

### Sample Size and Power Analysis

Sample size calculations were run to determine the number of participants required in the study to have an 80% chance of detecting a 0.3 standard deviation effect of the intervention based on the results of the outcome measures. Based on these criteria, we would need complete data from 180 participants in each arm of the study. Attrition and loss to follow-up is considered to be low, in the order of 25%.

As such, we would need to recruit 240 participants to each arm of the study. However, as this is a feasibility study, we will recruit 40 participants to each arm of the trial and evaluate the procedure used, time, and feasibility of achieving a larger sample for any future larger-scale study.

### Outcomes, Confounders, and Measurement Instruments

#### Primary outcome: Engagement in education and learning

We will use a short form of the validated Attitudes to School Survey developed by Khoo and Ainley (2005) and used in the *Longitudinal Study of Australian Children*. The survey has been used and referred to by the authors as a measure of school engagement as it aims to measure aspects of the complex relationship between engagement, attitudes, and motivation in terms of their influence on intentions to participate in school and schoolwork. The survey includes a primary and a secondary school version each consisting of 30 statements that cover 5 domains: students’ general satisfaction with school, their motivation, their attitudes toward their teachers, their views on the opportunities their school provides, and their sense of achievement. Students are asked to indicate their level of agreement on a 4-point Likert scale ranging from *strongly agree* to *strongly disagree*.

- *General Satisfaction*, sometimes called positive affect, reflects favorable feelings about school as whole. A typical item is *my school is a place where I really like to go each day*.
- *Motivation* represents a sense of self-motivation in learning and that learning is enjoyable for its own sake. A typical item is *my school is a place where I get excited about the work we do*.
- *Achievement* reflects a sense of confidence in one’s ability to be successful in school. A typical item is *my school is a place where I am good at schoolwork*.
- *Opportunity* represents a belief in the relevance of schooling for the future. A typical item is *my school is a place where what I learn will be useful*.
- *Teachers* refers to a feeling about the adequacy of the interaction between teachers and students. A typical item is *my school is a place where my teacher listens to what I say*.

We have developed a short form (15 statement) version, anticipating that the full version would be potentially too burdensome for the hospitalized and chronically ill child or young person. Items were chosen on the basis of their face validity/perceived appropriateness by the author, who had more than three years’ experience working with hospital-based teachers/educators and young people at the Royal Children’s Hospital in Melbourne, Australia, 2012–2016.

There are two ways to score the items: (a) calculate the total level of agreement and (b) calculate a scale score for the domains and survey as a whole. We will use the latter method as we are interested in the overall mean values of both the intervention and the control groups and related standard deviations.

#### Secondary outcome: Quality of life

There has been an increased recognition of the importance of measuring the quality of life of children and young people with chronic health conditions as a standardized way of measuring treatment effectiveness within the child’s functional context (Haverman, Limperg, Young, Grootenhuis, & Klaassen, 2017; Silva, Crespo, Carona, Bullinger, & Canavarro, 2015; Wright & Majnemer, 2014). Measuring quality of life in a validated and standardized way allows for evaluating both intervention effectiveness and cost effectiveness (Aledort et al., 2012).

The Pediatric Quality of Life (PedsQL™; Farias Queiroz, Costa Amorim, Zandonade, & Monteiro de Barros Miotto, 2015)) inventory is a widely used and validated modular measurement of health-related quality of life. It has been designed to measure the core dimensions of health as delineated by the World Health Organization, including school functioning. The 23-item measure covers four domains: physical functioning, emotional functioning, social functioning, and school functioning. It has both self-report and parent proxy report versions, and can be scored so as to obtain a total scale score or summary scores for physical health and psychosocial health. However, scale scores for each of the four domains have been used in previous research of children and young people with chronic illnesses (Farias Queiroz et al., 2015). There are age specific versions for 5–7 yo, 8–12 yo, and 13–18 yo children and young people.

### Confounders

Family socio-economic characteristics are well known as predictors (although not a prescription) of how well children and young people perform at school, both in terms of engagement and academic achievement (Daraganova, 2012; Hancock et al., 2013). We will, therefore, collect information on and control for the impacts of family socio-economic position; a composite measure made up of information on the mother’s highest level of education, parental income, and occupation type/status (Daraganova, 2012).

Parental involvement in the child’s education is also understood to be a predictor of the child’s educational engagement and achievement, and has also been shown to mediate the influence of both family income and maternal education (Altschul, 2012). For this feasibility study, we will use three measures of parental school engagement that have been used in the LSAC:

*How often do you and study child talk about his/her school activities?*

(a) Daily; (b) A few times a week; (c) About once a week; (d) A few times a month; (e) Rarely or never.

*During this school year, how often did someone in this household help the child with his/her homework?*

(a) 5 or more days a week; (b) 3 or 4 days a week; (c) 1 or 2 days a week; (d) Less than once a week; (e) Never.

*In the last 12 months, how many times have you contacted the school about the child’s academic program for this year?*

(a) Not at all; (b) Once or twice; (c) Three or four times; (d) More than four times.

Parents/carers of children and young people with a chronic health condition experience higher levels of psychological stress, anxiety, and depression than parents with such a condition (Barnett, Giallo, Kelaher, Goldfeld, & Quach, 2018; Muscara et al., 2015). In addition, parents’ mental health and parenting style are known to be important predictors of a child’s academic performance and engagement in school, education, and learning (Barnett et al., 2018). We will use the Depression Anxiety Stress Scales (DASS-21; Lovibond & Lovibond, 1995), which is a validated measure of anxiety, stress, and depression. The utility of the measure is enhanced by the provision of normative data.

### Blinding

Blinding refers to keeping study participants and personnel “unaware” of which study group they belong to (i.e., intervention group or no-intervention/control group). Blinding in controlled studies is considered an important strategy for minimizing the potential of any performance or detection bias. However, blinding of personnel and participants is often difficult when evaluating psychosocial interventions (Montgomery et al., 2013), and blinding of both groups is considered not achievable in the current study.

### Data Collection

#### Baseline data

Baseline data will be collected by the lead researcher or hospital-based educator at the time of student recruitment to the study or within one week of recruitment. It will consist of basic demographic data of the young person (age, gender, school grade/level, type of chronic illness, cultural identity, main language spoken at home) and of the parent (socio-economic position, involvement in child’s education, and psychological stress). We will also collect baseline data on the primary and secondary outcomes using the measures described above.

#### 3-month and 6-month data

We will collect primary and secondary outcome data at both the 3- and 6-month time points post the time of baseline data collection.

In addition, we will collect information on the number and type of education support activities (as described in the program logic model; see Figure 2) that were received by the young person, his or her parent/s, and the school for both the intervention and control groups.

#### Stakeholder interviews

Aim 4 of the study is to obtain feedback from stakeholders about the feasibility of conducting a larger-scale trial based on the experience and learnings of the feasibility study. The lead researcher will conduct semi-structured interviews with two key stakeholders who have been involved in the study at each of the five sites in order to obtain information about their experience of the implementation of the feasibility study and learnings.

### Data Analysis

The primary and secondary outcomes form the dependent variables in this research. The independent variables include being in receipt of the intervention or not being in receipt of the intervention (i.e., membership of either the intervention or the control group).

Quantitative data will be analyzed descriptively. Continuous or scale data will be analyzed using analysis of variance (ANOVA), and we will report group means and standard deviations. We will control for confounders as previously described. We will use the data and variables of the number and type of education support activities as a measure of dose and perform sensitivity analysis. Qualitative data obtained from stakeholder interviews will be analyzed thematically.

## Discussion and Conclusion

This protocol paper provides information about how a feasibility study could be undertaken to obtain robust information about the effectiveness of an education support program for hospitalized students with chronic health conditions. While the protocol was developed to evaluate the effectiveness of the education support program at the Royal Children’s Hospital in Australia, the study design is potentially replicable for evaluating other hospital-based education support programs for children and young people with chronic health conditions.

To date, evidence of effectiveness of education support programs for this group of children and young people is dominated by qualitative research, case studies, and expert opinion. More robust evidence is needed. Importantly, this feasibility study protocol tackled some important questions or considerations, including developing an answerable research question, choosing a controlled study design that compares the outcomes of both an intervention group and a well-matched non-intervention or control group, eligibility criteria, important and validated outcome measures, and how data should be analyzed and reported. The use of relevant and validated outcome measures, in particular quality of life, and consistent reporting of results using group means and standard deviations, is particularly important for future research in this field. Doing so would allow for comparing and even pooling results from different studies, thus providing even more robust evidence of the effectiveness of education support programs. To date, this has not been possible due to significant inconsistencies in these areas across the small number of controlled studies in this field.

Feasibility studies are considered useful as preparation for the conduct of a larger controlled evaluation of complex interventions (Thabane et al., 2016) by providing information about the feasibility of a main or larger study, with the goal of reducing uncertainty, and thereby increasing the chance of a successful larger study. As such, the current feasibility study includes a qualitative component to review and assess important aspects of the implementation of the trial, including recruitment, administering the measurement tools, and follow-up with trial participants. Such experience and learnings would be useful for preparing for any future larger study.
